# To Breathe or Not to Breathe: Spontaneous Ventilation During Thoracic Surgery in High-Risk COPD Patients—A Feasibility Study

**DOI:** 10.3390/jcm14228244

**Published:** 2025-11-20

**Authors:** Matyas Szarvas, Csongor Fabo, Gabor Demeter, Adam Oszlanyi, Stefan Vaida, Jozsef Furak, Zsolt Szabo

**Affiliations:** 1 Department of Anesthesiology and Intensive Therapy, University of Szeged, H-6720 Szeged, Hungary; matyasszarvas97@gmail.com (M.S.);; 2 Department of Anesthesiology and Intensive Care, Bács-Kiskun County Teaching Hospital, H-6000 Kecskemet, Hungary; 3 Department of Surgery, St. Elisabeth Hospital, H-5100 Jászberény, Hungary; stefan_vaida@yahoo.com; 4 Department of Surgery, University of Szeged, H-6720 Szeged, Hungary; 5 Doctoral School of Multidisciplinary Medicine, University of Szeged, H-6720 Szeged, Hungary

**Keywords:** chronic obstructive pulmonary disease, video-assisted thoracoscopic surgery, spontaneous ventilation, NIVATS, SVI lobectomy

## Abstract

**Background**: Spontaneous ventilation with intubation (SVI) during video-assisted thoracoscopic surgery (VATS) has been introduced as a hybrid technique that combines the physiological benefits of spontaneous breathing with the safety of a secured airway. However, its application in patients with chronic obstructive pulmonary disease (COPD) remains controversial due to concerns about hypercapnia, hypoxemia, and dynamic hyperinflation. To date, no study has directly compared COPD and non-COPD patients undergoing VATS lobectomy under SVI using identical anesthetic and surgical protocols. **Methods**: A prospective observational study was conducted between January 2022 and December 2024 at a single tertiary thoracic surgery center. A total of 36 patients undergoing elective VATS lobectomy with SVI were included and divided into two groups: COPD (*n* = 17) and non-COPD (*n* = 19), based on GOLD criteria. All patients were intubated with a double-lumen tube and allowed to maintain spontaneous ventilation during one-lung ventilation (OLV) after recovery from neuromuscular blockade. Arterial blood gas (ABG) samples were collected at four predefined time points (T1–T4), and intraoperative respiratory parameters, hemodynamics, spontaneous ventilation time, and spontaneous ventilation fraction (SpVent%) were recorded. Postoperative outcomes, including ICU stay, complications, and conversion to controlled ventilation, were analyzed. Statistical comparisons were performed using *t*-test, Mann–Whitney U test, chi-square test, and ANCOVA with adjustment for age, sex, BMI, and FEV_1_%. **Results**: All 36 procedures were successfully completed under SVI without conversion to controlled mechanical ventilation or thoracotomy. Baseline demographics were comparable between COPD and non-COPD patients regarding age (68.4 ± 6.9 vs. 67.8 ± 7.1 years; *p* = 0.78) and BMI (27.1 ± 4.6 vs. 26.3 ± 4.2 kg/m^2^; *p* = 0.56), while pulmonary function was significantly lower in COPD patients (FEV_1_/FVC 53.8% (IQR 47.5–59.9) vs. 82.4% (78.5–85.2); *p* < 0.001). The duration of spontaneous ventilation was significantly longer in the COPD group (82 ± 14 min vs. 58 ± 16 min; *p* < 0.001), and remained significant after ANCOVA adjustment (β = +23.7 min; *p* = 0.001). The SpontVent% was higher in COPD patients (80% [70–90] vs. 60% [45–80]), showing a trend toward significance (*p* = 0.11). Intraoperative permissive hypercapnia was well tolerated: peak PaCO_2_ levels at T3 were higher in COPD (52 ± 6 mmHg) than in non-COPD patients (47 ± 5 mmHg; *p* = 0.06), without pH dropping below 7.25 in either group. No significant differences were observed in mean arterial pressure, oxygen saturation, ICU stay (1.1 ± 0.4 vs. 1.0 ± 0.5 days; *p* = 0.48), or postoperative complication rates (*p* = 0.67). All patients were extubated in the operating room. **Conclusions**: Intubated spontaneous ventilation during VATS lobectomy is feasible and safe in both COPD and non-COPD patients when performed by experienced teams. COPD patients, despite impaired baseline lung function, were able to maintain spontaneous breathing for significantly longer periods without developing severe hypercapnia, acidosis, or hemodynamic instability. These findings suggest that SVI may represent a lung-protective alternative to fully controlled one-lung ventilation, particularly in hypercapnia-adapted COPD patients. Further multicenter studies are warranted to validate these results and define standardized thresholds for CO_2_ tolerance, patient selection, and intraoperative monitoring during SVI.

## 1. Introduction

Video-assisted thoracoscopic surgery (VATS) has become the preferred approach for anatomical lung resections due to its association with reduced postoperative pain, fewer pulmonary complications, and shorter hospital stay compared with open thoracotomy [1,2,3]. Despite these advantages, VATS still traditionally requires one-lung ventilation (OLV) using a double-lumen tube (DLT) or bronchial blocker to collapse the operative lung and maintain adequate oxygenation in the dependent lung [4,5,6]. However, OLV under controlled mechanical ventilation can lead to volutrauma, barotrauma, atelectrauma, and ventilator-induced lung injury (VILI), particularly in patients with pre-existing pulmonary impairment such as chronic obstructive pulmonary disease (COPD) [5,7,8,9].

In order to reduce the adverse physiological consequences of intubation and controlled ventilation, non-intubated VATS (NIVATS) techniques have emerged. These approaches avoid tracheal intubation and allow patients to maintain spontaneous respiration under regional anesthesia or sedation [2,9,10,11]. NIVATS has been associated with reduced postoperative inflammatory response, shorter recovery times, and fewer airway complications [6,12]. However, this technique may be limited by risks such as hypoventilation, hypercapnia, mediastinal shift, cough reflex, and airway obstruction, especially in complex resections or patients with reduced pulmonary reserve [13,14,15].

To combine the advantages of spontaneous ventilation with the safety of a secured airway, spontaneous ventilation with intubation (SVI) has been introduced as a hybrid technique. In this approach, a double-lumen tube is placed to facilitate selective lung collapse, but patients maintain spontaneous breathing for part or all of the OLV period, with mechanical ventilation only as backup support [11,16,17,18,19]. This technique preserves diaphragmatic motion, reduces airway pressures, and may improve ventilation-perfusion matching, while still ensuring airway protection and immediate conversion to full mechanical ventilation if needed [16,17,20].

COPD patients present unique challenges during thoracic surgery: airflow limitation, dynamic hyperinflation, air trapping, pulmonary hypertension, and chronic CO_2_ retention make them particularly vulnerable to the adverse effects of controlled OLV and mechanical ventilation [15,20,21,22,23]. Hypercapnia during OLV is often tolerated (“permissive hypercapnia”), but excessive CO_2_ retention can impair right ventricular function, increase pulmonary vascular resistance, and induce respiratory acidosis [24,25,26]. At the same time, COPD patients may actually tolerate moderate hypercapnia better than non-COPD patients due to chronic adaptation, raising the question of whether spontaneous ventilation could provide physiological benefits by avoiding high airway pressures and promoting more natural breathing mechanics [27,28,29].

However, the current literature predominantly reports single-arm feasibility studies of NIVATS and SVI in COPD patients only, without direct comparison to non-COPD populations undergoing the same procedure under identical anesthetic conditions [23,26,30,31,32]. To date, no study has systematically compared the intraoperative tolerance of spontaneous ventilation, gas exchange (PaCO_2_, PaO_2_), and perioperative outcomes between COPD and non-COPD patients undergoing VATS lobectomy with SVI. Several studies have suggested that maintaining spontaneous breathing during thoracic surgery may reduce ventilator-induced lung injury, improve hemodynamic stability, and shorten postoperative recovery, but the degree to which these benefits apply specifically to COPD patients compared with non-COPD populations remains unclear [33,34,35,36,37,38]. Moreover, it is not known whether COPD patients are more likely to sustain a longer percentage of spontaneous ventilation during one-lung ventilation (SVI%), or whether they experience more pronounced hypercapnia or respiratory acidosis than non-COPD patients under the same anesthetic protocol. Recent advances in anesthetic monitoring, permissive hypercapnia strategies, and individualized airway management have made SVI a viable alternative to fully controlled ventilation, yet its physiological effects in different pulmonary phenotypes are still under investigation [19,32,39,40,41,42].

Therefore, there is a need for comparative clinical data assessing whether COPD patients can tolerate prolonged spontaneous ventilation during VATS lobectomy and whether this approach results in different intraoperative gas exchange patterns, hemodynamic responses, and postoperative outcomes compared with patients without COPD.

This study aimed to fill this gap by conducting a two-cohort analysis of patients undergoing VATS lobectomy with spontaneous ventilation and intubation (SVI): one group with confirmed COPD and a matched non-COPD control group. Specifically, we sought to (1) evaluate the feasibility and safety of maintaining spontaneous ventilation during one-lung ventilation in COPD patients, (2) compare intraoperative CO_2_ levels, respiratory acidosis, and hemodynamics between COPD and non-COPD patients, and (3) assess perioperative outcomes including conversion to mechanical ventilation, postoperative complications, and length of hospital stay [12,16,19,20,22,34].

We hypothesized that COPD patients, due to their chronically elevated baseline PaCO_2_ and adaptive ventilatory physiology, would tolerate permissive hypercapnia and maintain spontaneous ventilation for a comparable or even longer proportion of operative time than non-COPD patients, without an increased rate of complications [12,20,23,27,42].

## 2. Materials and Methods

### 2.1. Study Design and Patient Selection

This prospective observational study was conducted at a single tertiary thoracic surgery center between January 2022 and December 2024. All procedures were performed by the same anesthesia and surgical team experienced in spontaneous ventilation techniques [11,12,16]. The study protocol was approved by the Institutional Review Board (Permission No. 4703/2020.01.20; Chairperson: Prof. Tibor Wittmann), and written informed consent was obtained from all patients.

Patients undergoing elective video-assisted thoracoscopic surgery (VATS) lobectomy under general anesthesia were evaluated for inclusion. Two groups were formed: patients with chronic obstructive pulmonary disease (COPD group, *n* = 17) and a control group without COPD (non-COPD group, *n* = 19). COPD was diagnosed according to the Global Initiative for Chronic Obstructive Lung Disease (GOLD) criteria, defined as a post-bronchodilator FEV1/FVC ratio < 70% with compatible clinical history and imaging findings [4].

Both groups underwent the same preoperative assessments, including pulmonary function testing (FEV1, FVC, FEV1/FVC%), arterial blood gas analysis, chest CT, electrocardiography, transthoracic echocardiography when indicated, and evaluation according to the European Society of Thoracic Surgeons (ESTS) guidelines for minimally invasive lobectomy [8,11].

Inclusion criteria:

- Age ≥ 18 years
- ASA physical status I–III
- Lung tumor ≤ 7 cm with N0 or N1 stage (ESTS criteria)
- Feasible for VATS lobectomy with double-lumen intubation and selective one-lung ventilation

Exclusion criteria:

- Locally advanced lung cancer (T4 or N2 disease)
- Prior contralateral lobectomy
- Hemodynamic instability, uncontrolled arrhythmia, or severe hypoxemia (PaO_2_ < 60 mmHg on room air)
- Morbid obesity (BMI > 35 kg/m^2^)
- Severe pulmonary hypertension (systolic pulmonary artery pressure > 50 mmHg)
- ASA physical status ≥ IV

Baseline demographic, clinical, and functional data were recorded, including age, sex, body mass index (BMI), smoking history (if available), ASA status, comorbidities (hypertension, coronary artery disease, diabetes mellitus), pulmonary function (FEV1% predicted, FEV1/FVC), and GOLD stage for COPD patients.

### 2.2. Anesthetic Management

All patients underwent general anesthesia with planned spontaneous ventilation during one-lung ventilation (OLV), following our previously published SVI protocol [5,10,11,19].

Standard intraoperative monitoring included three-lead electrocardiography (ECG), pulse oximetry, invasive radial arterial blood pressure, and bispectral index (BIS) monitoring using a BIS Vista™ monitor (Medtronic, Minneapolis, MN, USA).

Anesthesia was induced with intravenous fentanyl (1–1.5 µg/kg; Richter Gedeon Nyrt., Budapest, Hungary) and propofol administered via a target-controlled infusion (TCI) system based on the Schnider model, using a Perfusor^®^ Space infusion pump (B. Braun Melsungen AG, Melsungen, Germany). The target effect-site concentration was set between 4–6 µg/mL to maintain a BIS value between 40 and 60 [17,40].

Neuromuscular blockade was facilitated with mivacurium chloride (Mivacron^®^, GlaxoSmithKline, Brentford, UK) to allow double-lumen tube (DLT) intubation. Intubation was confirmed by flexible bronchoscopy using an Ambu^®^ aScope™ 4 Broncho Regular (Ambu A/S, Ballerup, Denmark).

Following positioning and thoracic access, intercostal nerve blocks, paravertebral blockade, and intrathoracic vagal nerve infiltration with 0.5% bupivacaine (Bupivacainum, Egis Pharmaceuticals Plc., Budapest, Hungary) were administered to provide analgesia and suppress the cough reflex [11,14,15].

After recovery from neuromuscular blockade, spontaneous breathing was allowed to resume while the DLT remained in place, enabling selective one-lung spontaneous ventilation.

During spontaneous ventilation, oxygenation was maintained with an FiO_2_ of 0.4–1.0, a positive end-expiratory pressure (PEEP) of 3–5 cmH_2_O to the dependent lung, and pressure support ventilation of 10–16 cmH_2_O if tidal volumes dropped below 3–4 mL/kg [5,19,25]. Care was taken to avoid excessive airway pressures (>20 cmH_2_O) to preserve diaphragmatic motion.

Conversion to controlled mechanical ventilation was initiated in cases of persistent hypoxemia (SpO_2_ < 90% for >2 min), severe hypercapnia (PaCO_2_ > 80 mmHg with pH < 7.20), hemodynamic instability, or uncontrollable cough or mediastinal shift [11,18,34,43].

Postoperatively, patients were transferred to the post-anesthesia care unit (PACU). Analgesia consisted of intravenous paracetamol (Perfalgan^®^, Bristol Myers Squibb, Rueil-Malmaison, France), non-steroidal anti-inflammatory drugs (Dexalgin^®^, Berlin-Chemie AG, Berlin, Germany), and patient-controlled analgesia using low-dose opioids if the Visual Analog Scale (VAS) pain score exceeded 3. Epidural or paravertebral catheters were not used to avoid interference with respiratory drive during SVI [16,35]. Patients were discharged from the PACU after achieving an Aldrete score ≥ 9.

### 2.3. Arterial Blood Gas Sampling

Arterial blood gas (ABG) analysis was performed at four standardized time points to assess gas exchange and acid–base status during spontaneous ventilation and one-lung ventilation, following previously published protocols on intraoperative CO_2_ monitoring and permissive hypercapnia in thoracic anesthesia [5,24,25,26,28]:

- T1: After radial arterial catheter insertion before induction, on room air (FiO_2_ = 0.21)
- T2: 15 min after initiation of one-lung spontaneous ventilation and completion of vagal block
- T3: 15 min after completion of lung resection while still under spontaneous ventilation
- T4: 30 min after arrival in the PACU, with supplemental oxygen (FiO_2_ = 0.5)

ABG measurements were performed using a Cobas b 123 POC system (Roche Diagnostics GmbH, Mannheim, Germany). At each time point, PaO_2_, PaCO_2_, pH, bicarbonate (HCO_3_^−^), lactate, and SaO_2_ were recorded.

Permissive hypercapnia was considered acceptable if pH ≥ 7.25 and systolic blood pressure remained stable, consistent with current recommendations for lung-protective anesthesia [28,29,42,44].

### 2.4. Surgical Technique

All procedures were performed using a standardized uniportal VATS technique by the same surgical team experienced in minimally invasive thoracic surgery and spontaneous ventilation protocols [8,11,16,19].

Patients were placed in the lateral decubitus position, and a single 3–4 cm incision was made in the 5th intercostal space along the anterior axillary line.

Selective one-lung ventilation was maintained via the double-lumen tube (Broncho-Cath™, Medtronic, Minneapolis, MN, USA) to allow collapse of the operative lung, while spontaneous ventilation was preserved in the dependent lung.

Thoracoscopic access was achieved using a 30° thoracoscope (Karl Storz SE & Co. KG, Tuttlingen, Germany). CO_2_ insufflation was avoided to prevent mediastinal shift or impaired venous return [13,15,17].

Standard anatomical lobectomy was performed according to ESTS consensus guidelines, with dissection of the pulmonary artery, vein, fissures, and bronchus using Endo GIA™ staplers (Medtronic, Minneapolis, MN, USA).

Systematic mediastinal lymph node dissection was carried out in all patients.

At the end of the procedure, an underwater seal chest drain (Atrium Ocean™, Getinge AB, Gothenburg, Sweden) was placed, and the lung was re-expanded under pressure support if required. All patients were extubated in the operating room unless hemodynamic instability or severe respiratory acidosis was present. 

Data were organized using Microsoft Excel 2021 (Microsoft Corp., Redmond, WA, USA) and analyzed with IBM SPSS Statistics for Windows, Version 29.0 (IBM Corp., Armonk, NY, USA). Figures were generated with GraphPad Prism, Version 10.1 (GraphPad Software, San Diego, CA, USA).

Generative AI tools were used solely for language editing and formatting support during manuscript preparation, without influencing the scientific content, data, or interpretations. 

## 3. Results

### 3.1. Baseline Characteristics

A total of 36 patients undergoing VATS lobectomy under intubated spontaneous ventilation were included in the final analysis, comprising 17 COPD and 19 non-COPD individuals. Baseline demographic and functional characteristics are summarized in Table 1, while comorbidities and preoperative clinical conditions are detailed in Table 2.

There were no significant differences between groups in age (COPD: 68.4 ± 6.9 vs. non-COPD: 67.8 ± 7.1 years; *p* = 0.78), sex distribution (71% vs. 68%; *p* = 0.84), body mass index (27.1 ± 4.6 vs. 26.3 ± 4.2 kg/m^2^; *p* = 0.56), or ASA physical status (*p* = 0.64).

As expected, preoperative lung function was significantly impaired in COPD patients, with markedly lower FEV_1_/FVC ratios and FEV_1_% predicted values compared with non-COPD individuals (*p* < 0.001). GOLD staging for COPD severity is presented in Table 1.

**Figure 1:**
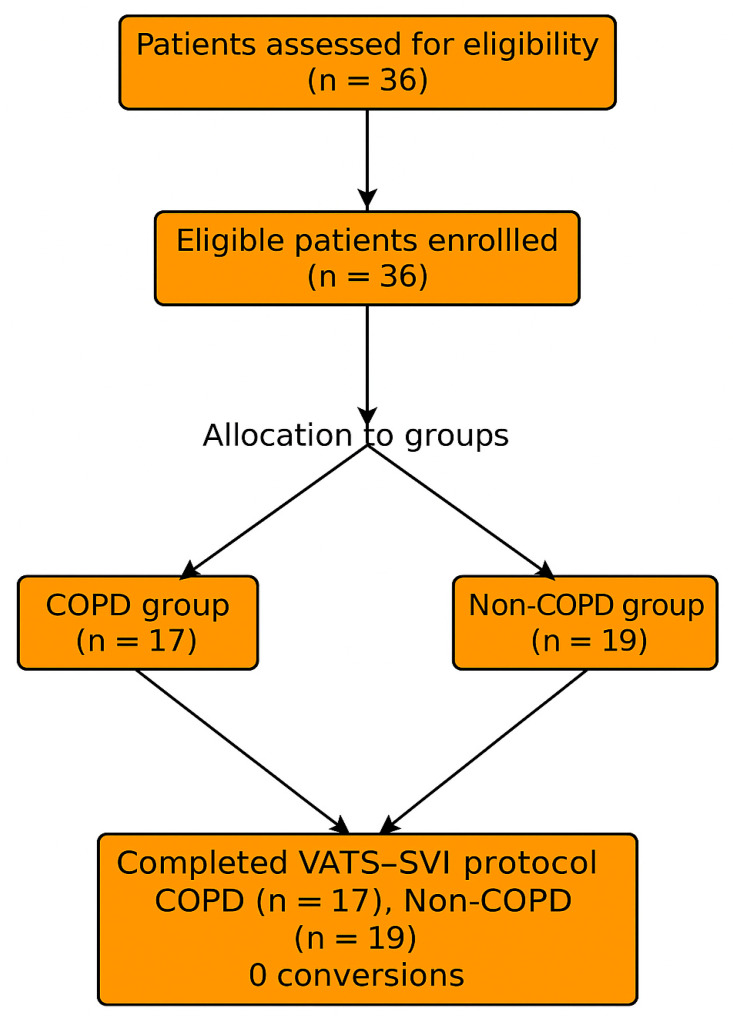
Illustration of the patient selection process, exclusion criteria, and final allocation to the COPD (*n* = 17) and non-COPD (*n* = 19) groups.

The prevalence of major comorbidities such as hypertension, coronary artery disease, diabetes mellitus, arrhythmia, and chronic heart failure did not differ significantly between groups (all *p* > 0.05; Table 2). No patient in either group required long-term oxygen therapy or home non-invasive ventilation preoperatively (Figure 1).

### 3.2. Primary Outcome Spontaneous Ventilation Fraction (SVI%)

The median proportion of spontaneous ventilation during one-lung ventilation (SVI%) was higher in the COPD group compared with non-COPD patients (80% (IQR 70–90) vs. 60% (IQR 45–80)); however, this difference did not reach statistical significance (*p* = 0.11; Table 3). The effect size indicated a small-to-moderate difference between groups (Cliff’s δ = 0.25).

After adjustment for age, sex, BMI, FEV_1_% predicted, and smoking status using analysis of covariance (ANCOVA), the group effect remained positive (β = +8.6%, 95% CI −3.1 to 20.3), but was still not statistically significant (*p* = 0.13).

**Figure 2:**
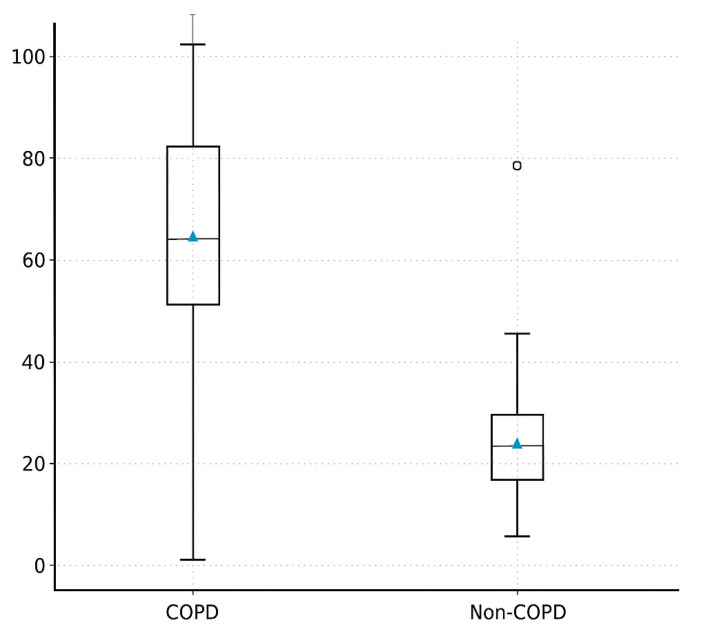
Demonstrates the distribution of SVI% between the COPD and non-COPD groups using a box-and-whisker plot, visually highlighting the higher median values and wider interquartile range in COPD patients. Box plots represent the distribution of spontaneous ventilation percentages in both groups. The central line indicates the median, and the box shows the interquartile range (IQR); whiskers represent the minimum and maximum values, and outliers are displayed as circles. The triangle (▲) indicates the mean value.

A higher proportion of COPD patients achieved prolonged spontaneous ventilation (SVI ≥ 70% of the OLV period) compared with non-COPD patients (64.7% vs. 42.1%), although this did not reach statistical significance (*p* = 0.19) (Figure 2).

### 3.3. Duration of Spontaneous Ventilation (Minutes)

The absolute duration of spontaneous ventilation was significantly longer in COPD patients compared with non-COPD individuals (82 ± 14 min vs. 58 ± 16 min; *p* < 0.001; Table 3). This difference corresponded to a large effect size (Cohen’s d = 0.86), indicating a strong discriminatory power in favor of the COPD group.

Importantly, this association remained significant after adjustment for age, sex, BMI, smoking status, and FEV_1_% predicted using ANCOVA (β = +23.7 min; 95% CI 10.8–36.6; *p* = 0.001).

**Figure 3:**
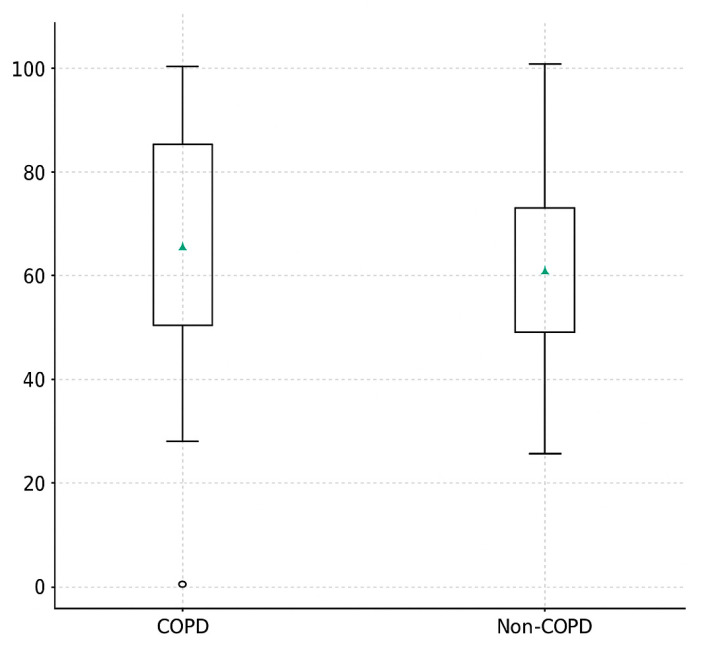
Spontaneous Ventilation Percentage in COPD and Non-COPD patients. Box plots represent the distribution of spontaneous ventilation percentages in both groups. The central line indicates the median, and the box shows the interquartile range (IQR); whiskers represent the minimum and maximum values, and outliers are displayed as circles. The triangle (▲) indicates the mean value.

No patient in either group required complete conversion to controlled mechanical ventilation, and none were excluded due to inability to maintain adequate oxygenation or hemodynamic stability (Figure 3).

### 3.4. Intraoperative Physiology and Gas Exchange

Intraoperative hemodynamics remained stable in both groups throughout one-lung ventilation. There were no significant differences in mean arterial pressure (COPD: 82 ± 9 mmHg vs. non-COPD: 84 ± 8 mmHg; *p* = 0.48), heart rate, or peripheral oxygen saturation (all *p* > 0.05; Table 3). No episodes of severe desaturation (SpO_2_ < 90%) or hemodynamic collapse were recorded.

End-tidal CO_2_ (ETCO_2_) values were higher in the COPD group (median 49 mmHg IQR 46–53]) compared with non-COPD patients (44 mmHg [41,42,43,44,45,46,47]; *p* = 0.06), reflecting permissive hypercapnia associated with spontaneous ventilation.

Arterial blood gas analysis demonstrated a controlled and reversible rise in PaCO_2_ during one-lung ventilation in both groups. PaCO_2_ values peaked at T2 (15 min after initiation of spontaneous ventilation) and T3 (end of lobectomy), with greater elevation in COPD patients; however, pH remained above 7.25 in all cases, and no patient met criteria for intervention or conversion to controlled mechanical ventilation (Table 4).

No conversion to full mandatory ventilation was required in either group, and no patient experienced hypercapnia-related arrhythmias, hypotension, or surgical interference due to diaphragmatic movement.

### 3.5. Postoperative Outcomes

All patients were successfully extubated in the operating room. No reintubation, unplanned ICU admission, or 30-day mortality occurred in either group. Postoperative outcomes are summarized in Table 5.

The median ICU stay was similar between COPD and non-COPD patients (1.1 ± 0.4 vs. 1.0 ± 0.5 days; *p* = 0.48), and no significant differences were observed in total hospital length of stay (6.3 ± 1.5 vs. 5.9 ± 1.6 days; *p* = 0.42). Chest tube duration did not differ significantly (3.7 ± 1.2 vs. 3.4 ± 1.1 days; *p* = 0.36).

The overall postoperative complication rate was comparable (COPD 23.5% vs. non-COPD 21.1%; *p* = 0.67), with most complications classified as Clavien–Dindo grade I–II (prolonged air leak, mild atelectasis, transient atrial fibrillation). No major cardiopulmonary events, pneumonia, or need for mechanical ventilatory support were recorded.

## 4. Discussion: Main Findings + Feasibility

This prospective analysis demonstrates that intubated spontaneous ventilation (SVI) during VATS lobectomy is feasible and physiologically safe in high-risk COPD patients [11,12,16,20]. Despite their limited pulmonary reserve, COPD patients maintained spontaneous ventilation significantly longer than non-COPD controls, without deterioration in gas exchange or hemodynamic stability. These findings suggest that SVI can be successfully applied in carefully selected COPD patients as part of a minimally invasive anesthetic strategy.

Our results show that COPD patients achieved a significantly longer duration of spontaneous ventilation (82 ± 14 vs. 58 ± 16 min, *p* < 0.001), while the relative proportion of SVI during one-lung ventilation was higher but did not reach statistical significance (80% vs. 60%, *p* = 0.11). Importantly, no conversion to controlled mechanical ventilation was required in either group, and no episode of severe hypoxemia, acidosis, or hemodynamic instability was observed. This confirms not only the feasibility but also the physiological tolerability of SVI in a population conventionally considered high-risk for dynamic airway collapse and hypercapnic sensitivity [12,13,19,22,43].

To our knowledge, this is the first study directly comparing COPD and non-COPD patients undergoing VATS lobectomy with intubated SVI, using standardized ABG measurements at four time points and quantifying SVI both as a percentage and absolute duration of one-lung ventilation. Previous studies have primarily focused on non-intubated approaches or single-arm COPD cohorts without direct control groups [3,11,16,23,30]. These data provide quantitative evidence that COPD patients—when appropriately selected—can tolerate and even benefit from the preservation of spontaneous respiratory effort during thoracic surgery [11,19,34].

### 4.1. Feasibility and Safety in the Context of Existing Literature

Several studies have reported that spontaneous ventilation during thoracic surgery can reduce peak airway pressures, improve ventilation–perfusion matching, and enhance postoperative recovery [2,3,11,16,18]. Most available evidence, however, comes from non-intubated VATS (NIVATS) techniques, where airway control may be limited, and conversion rates of 2–10% are reported in high-risk patients [15,21,24,35]. In contrast, intubated SVI combines the advantages of preserved diaphragmatic motion with the safety of double-lumen tube placement, enabling rapid transition to full mechanical ventilation if required [11,19,20,32].

Our findings extend this concept to COPD patients, a population often excluded from NIVATS studies due to concerns about dynamic hyperinflation, CO_2_ retention, or difficult airway management [13,14,23,31]. Notably, none of the COPD patients in our study required conversion to controlled mechanical ventilation or experienced severe hypoxemia (SpO_2_ < 90%) or hemodynamic instability. These results support the idea that when performed by experienced teams, SVI is not only technically feasible but clinically safe even in severely impaired pulmonary physiology.

Previous studies by Pompeo et al. and Gonzalez-Rivas et al. demonstrated the feasibility of non-intubated VATS in selected COPD cases, but reported conversion rates of up to 8–12% due to hypoventilation or mediastinal shift [1,3,22,24]. In contrast, our intubated-SVI approach resulted in a 0% conversion rate, suggesting that maintenance of airway control may improve procedural safety without negating the physiological benefits of spontaneous breathing [11,12,19,20].

### 4.2. Physiological Interpretation—Hypercapnia Tolerance and Adaptive Mechanisms

An intriguing finding of this study is that COPD patients sustained spontaneous ventilation for significantly longer periods than non-COPD patients. This may be partially explained by chronic physiological adaptation to hypercapnia and respiratory load. In COPD, long-standing CO_2_ retention induces renal bicarbonate retention, resulting in metabolic compensation and stabilization of blood pH despite elevated PaCO_2_ levels. This compensatory mechanism allows COPD patients to tolerate higher intraoperative PaCO_2_ values (50–70 mmHg) without developing clinically significant acidosis (pH remained ≥ 7.25 in all cases) [24,25,27,28,29].

Furthermore, chronic hypercapnia blunts the central chemoreceptor sensitivity to CO_2_, leading to a flatter ventilatory response curve. As a result, respiratory drive remains stable even at higher PaCO_2_ values, reducing the risk of apnea during anesthesia. This can explain why the COPD cohort in our study demonstrated longer spontaneous ventilation times and fewer episodes of ventilatory suppression compared with non-COPD patients [27,28,42].

Importantly, permissive hypercapnia during SVI did not compromise oxygenation or hemodynamic stability. Instead, PaCO_2_ elevation was moderate and reversible (Table 4), and no patient developed severe respiratory acidosis or required conversion to controlled ventilation [12,20,43].

Beyond passive tolerance, mild hypercapnia may exert protective biological effects. Experimental and clinical studies have shown that hypercapnic acidosis attenuates inflammatory cytokine release, reduces alveolar overdistension, and mitigates ventilator-induced lung injury (VILI) [9,18,42,45]. In this context, SVI may function as a self-regulating, lung-protective ventilation strategy where diaphragmatic motion is preserved, transpulmonary pressure swings are physiologic, and mechanical stress on the alveoli is reduced [11,19,36,37].

### 4.3. Protective and Minimally Invasive Ventilation Strategy

Traditional one-lung ventilation during thoracic surgery relies on controlled mechanical ventilation, which may generate high transpulmonary pressures, cyclic alveolar collapse, volutrauma, and shear stress—effects that are particularly harmful in emphysematous or heterogeneous COPD lungs [5,8,9,20]. In contrast, SVI preserves diaphragmatic motion and maintains more physiological ventilation–perfusion matching, thereby reducing alveolar overdistension and improving oxygenation without the need for high airway pressures [11,16,18,19].

Unlike non-intubated VATS (NIVATS), SVI maintains a secured airway via double-lumen intubation while still allowing the patient to generate negative intrathoracic pressures. This hybrid approach combines the safety of controlled airway management with the physiological advantages of spontaneous breathing [3,11,14,19,43]. It minimizes the risks of airway collapse, turbulent gas flow, and inadequate CO_2_ clearance while maintaining the ability to switch to mandatory ventilation if required.

Our findings reinforce the concept of “protective spontaneous ventilation”—a strategy where respiratory effort is preserved not to avoid intubation, but to prevent ventilator-induced lung injury (VILI) [19,36,37,46]. By avoiding deep muscle relaxation and high-pressure ventilation, mechanical stress on lung tissue is minimized. Furthermore, anesthetic depth can be reduced during SVI, resulting in decreased opioid and hypnotic requirements, faster emergence, and early postoperative mobilization [16,18,35].

This concept aligns with enhanced recovery after surgery (ERAS) principles, and may be particularly beneficial in high-risk patients with reduced respiratory reserve, chronic hypercapnia, or intolerance to conventional volume-controlled ventilation [19,22,34,47].

### 4.4. Clinical Implications and Future Directions

Clinically, our findings suggest that SVI can be safely implemented in appropriately selected COPD patients undergoing VATS lobectomy when managed by experienced anesthesiology and surgical teams [11,12,16,19,20]. Moderate hypercapnia (PaCO_2_ 50–70 mmHg) should be considered acceptable during SVI as long as arterial pH remains ≥ 7.25 and hemodynamic stability is preserved, consistent with the concept of permissive hypercapnia and lung-protective ventilation [26,28,29,42,44]. Our data indicate that maintaining spontaneous breathing may facilitate improved intraoperative lung mechanics without increasing perioperative risk.

These results support the integration of SVI into multimodal lung-protective strategies, particularly in patients with emphysema, hyperinflation, or limited tolerance to positive-pressure ventilation [18,19,34,37]. Future multicenter clinical trials with larger cohorts are required to validate these findings, define optimal thresholds for PaCO_2_, pH, and sedation depth, and determine whether SVI confers longer-term benefits in postoperative pulmonary function, inflammatory biomarkers, or recovery time [19,22,27,32].

Comparative studies between intubated SVI and non-intubated VATS could also help clarify the balance between airway safety and respiratory autonomy, potentially guiding patient-specific protocol selection [3,11,14,35].

### 4.5. Study Limitations

The main limitation of this study is the relatively small sample size, which may limit statistical power and generalizability. Although consistent trends and large effect sizes were observed for spontaneous ventilation time, the study may have been underpowered to detect differences in SVI% as a continuous variable. Additionally, this was a single-center, non-randomized study, and potential selection bias cannot be entirely excluded [12,20]. Patients with extremely severe COPD or preoperative ventilatory failure were not included, which limits extrapolation to end-stage disease [23].

Another limitation is the absence of long-term postoperative respiratory follow-up or chronic lung function assessment, meaning that our analysis focuses primarily on intraoperative feasibility and early outcomes. Future research should incorporate longitudinal functional testing, quality-of-life evaluation, and cost-effectiveness analysis [19,22,42].

### 4.6. Summary of Key Insights

- SVI is feasible and safe in COPD patients during VATS lobectomy.
- COPD patients maintain spontaneous breathing significantly longer, likely due to adaptive hypercapnia tolerance.
- SVI aligns with the concept of lung-protective ventilation and may reduce ventilator-induced lung injury.
- Further large-scale, randomized studies are needed to confirm these findings and standardize patient selection criteria and intraoperative SVI thresholds.

## 5. Conclusions

This prospective two-cohort study confirms that intubated spontaneous ventilation (SVI) during VATS lobectomy is safe and feasible not only in non-COPD patients but also in high-risk individuals with COPD. Despite significantly impaired baseline lung function, COPD patients were able to sustain spontaneous ventilation for longer periods than non-COPD patients, without experiencing clinically relevant hypoxemia, hypercapnia-induced acidosis, or hemodynamic instability. These findings suggest that SVI represents a viable alternative to fully controlled one-lung ventilation, even in patients traditionally considered at high anesthetic risk.

By preserving diaphragmatic motion and allowing permissive hypercapnia within physiological limits, SVI may function as a minimally invasive, lung-protective ventilation strategy, particularly beneficial in hypercapnia-adapted COPD physiology. Importantly, no conversions to controlled ventilation or major perioperative complications occurred in either group, reinforcing the safety profile of this approach when performed by experienced teams.

Future multicenter studies with larger sample sizes and long-term follow-up are required to validate these results, optimize patient selection criteria, and determine standardized thresholds for PaCO_2_, pH, and sedation depth during SVI.

## Figures and Tables

**Table 1:** Baseline demographic and pulmonary function characteristics of COPD and non-COPD patients.

Parameter	COPD (*n* = 17)	Non-COPD (*n* = 19)	*p* Value
Age (years)	68.4 ± 6.9	67.8 ± 7.1	0.78
Sex (M/F)	12/5	13/6	0.84
BMI (kg/m^2^)	27.1 ± 4.6	26.3 ± 4.2	0.56
ASA II/III (%)	47/53	53/47	0.64
Smoking history (%)	88	63	0.09
FEV_1_ (% pred)	61 ± 9	83 ± 8	<0.001 ***
FVC (% pred)	72 ± 10	95 ± 7	<0.001 ***
FEV_1_/FVC (%)	53.8 [47.5–59.9]	82.4 [78.5–85.2]	<0.001 ***

Note: Data are presented as mean ± SD, median (IQR), or *n* (%). Statistical comparison was performed using Stu-dent’s *t*-test or Mann–Whitney *U* test for continuous variables and chi-square or Fisher’s exact test for categorical variables. *** *p* < 0.001.

**Table 2:** Comorbidities and Preoperative Clinical Characteristics.

Comorbidity Category	Variable	COPD (*n* = 17)	Non-COPD (*n* = 19)	*p* Value
**Cardiovascular**	Hypertension	13 (76%)	12 (63%)	0.42
	Coronary artery disease	8 (47%)	6 (32%)	0.36
	Arrhythmia (AF or other)	4 (24%)	3 (16%)	0.55
	Heart failure (NYHA II–III)	3 (18%)	1 (5%)	0.23
**Metabolic**	Diabetes mellitus type II	5 (29%)	4 (21%)	0.57
	Dyslipidemia	6 (35%)	5 (26%)	0.53
	Obesity (BMI ≥ 30 kg/m^2^)	3 (18%)	2 (11%)	0.61
**Pulmonary**	Asthma or chronic bronchitis	5 (29%)	2 (11%)	0.17
	Pulmonary hypertension	3 (18%)	0 (0%)	0.06
	History of prior thoracic surgery	2 (12%)	1 (5%)	0.44
**Renal/Hepatic**	Chronic kidney disease	2 (12%)	1 (5%)	0.44
	Chronic liver disease	1 (6%)	0 (0%)	0.29
**Other**	Smoking (current/former)	15 (88%)	12 (63%)	0.09
	ASA physical status ≥ III	9 (53%)	9 (47%)	0.64

Data are presented as *n* (%). *p* values are calculated using chi-square or Fisher’s exact test.

**Table 3:** Intraoperative ventilatory parameters and postoperative outcomes in COPD vs. non-COPD patients undergoing VATS-SVI lobectomy.

Variable	COPD	Non-COPD	*p* Value
Spontaneous ventilation time (min)	82 ± 14	58 ± 16	<0.001 ***
Spontaneous ventilation fraction (%)	80 [70–90]	60 [45–80]	0.11
MAP (mmHg)	82 ± 9	84 ± 8	0.48
HR (bpm)	76 ± 10	78 ± 12	0.52
ETCO2 (mmHg)	49 [46–53]	44 [41–47]	0.06
PaO2 (mmHg)	108 ± 15	111 ± 14	0.42
Conversion to controlled ventilation (%)	0	0	—

Data are presented as mean ± SD, median (IQR), or n (%). Statistical comparison was performed using Student’s *t*-test or Mann–Whitney U test for continuous variables and chi-square or Fisher’s exact test for categorical variables. *** *p* < 0.001.

**Table 4:** Intraoperative blodd gas parameters.

Time Point	COPD PaCO_2_ (mmHg)	Non-COPD PaCO_2_	pH COPD	pH Non-COPD
T1 (Pre-op)	44.8 ± 5.2	39.6 ± 4.8	7.41 ± 0.03	7.43 ± 0.03
T2 (During SVI)	52.6 ± 6.7	46.3 ± 5.1	7.36 ± 0.04	7.39 ± 0.03
T3 (Post-resection)	55.8 ± 7.2	48.7 ± 5.5	7.34 ± 0.05	7.37 ± 0.04
T4 (PACU)	47.3 ± 5.9	42.5 ± 4.9	7.38 ± 0.03	7.40 ± 0.04

**Table 5:** Postoperative outcomes.

Outcome	COPD	Non-COPD	*p* Value
ICU stay (days)	1.1 ± 0.4	1.0 ± 0.5	0.48
Total hospital stay (days)	5.8 ± 1.7	5.6 ± 1.5	0.72
Postoperative complications (%)	12%	11%	0.67
Reintubation (%)	0	0	—
30-day mortality (%)	0	0	—

## Data Availability

The data presented in this study are available from the corresponding author upon reasonable request. The data are not publicly available due to ethical restrictions and patient confidentiality.
